# Gastrointestinal Adenocarcinoma Incidence and Survival Trends in South Australia, 1990–2017

**DOI:** 10.3390/cancers14020275

**Published:** 2022-01-06

**Authors:** Dominique Schell, Shahid Ullah, Mark E. Brooke-Smith, Paul Hollington, Marina Yeow, Christos S. Karapetis, David I. Watson, Stephen J. Pandol, Claire T. Roberts, Savio G. Barreto

**Affiliations:** 1 College of Medicine and Public Health, Flinders University, Adelaide, SA 5042, Australia; sche0142@flinders.edu.au (D.S.); shahid.ullah@flinders.edu.au (S.U.); paul.hollington@sa.gov.au (P.H.); chris.karapetis@sa.gov.au (C.S.K.); david.watson@flinders.edu.au (D.I.W.); 2 Flinders Medical Center, Division of Surgery and Perioperative Medicine, Flinders University, Adelaide, SA 5042, Australia; mark.brooke-smith@flinders.edu.au (M.E.B.-S.); marina.yeow2@sa.gov.au (M.Y.); 3 Flinders Health and Medical Research Institute, Flinders University, Adelaide, SA 5042, Australia; 4 Department of Medical Oncology, Flinders Medical Centre, Flinders University, Adelaide, SA 5042, Australia; 5 Cedars-Sinai Medical Center, Division of Digestive and Liver Diseases, Los Angeles, CA 90048, USA; stephen.pandol@cshs.org

**Keywords:** outcomes, morbidity, mortality, stomach, pancreas, colon

## Abstract

**Simple Summary:**

This study from South Australia using the state’s Cancer Registry data provides compelling evidence for a significant increase in the incidence of young-onset (18–50 years) gastrointestinal (oesophageal, stomach, colon and rectum, and pancreas) adenocarcinomas over the last three decades. The trend observed in the young cohort was not mirrored in older individuals >50 years. This increased incidence, though noted in both sexes, was more pronounced in males compared to females. Survival in the young-onset adenocarcinoma cohort was only seen in patients with colorectal cancers, but not oesophagus, stomach and pancreas. This study calls for a concerted effort aimed at determining the sociodemographic factors underlying this disturbing trend with the aim of developing preventative strategies.

**Abstract:**

Background & Aims: Globally, there has been a concerning rise in the incidence of young-onset cancers. The aim of this study was to provide trends in the incidence and survival of gastrointestinal adenocarcinomas (oesophagus, stomach, pancreas, and colorectal) in South Australia over a 27-year period. Methods: This is a cross-sectional analysis of a prospective longitudinal database including all cases of gastrointestinal adenocarcinomas prospectively reported to the South Australian (State) Cancer Registry from 1990 to 2017. Results: A total of 28,566 patients diagnosed with oesophageal, stomach, pancreatic, or colorectal adenocarcinoma between 1990 and 2017 were included in the study. While the overall incidence for gastrointestinal adenocarcinomas in individuals >50 years has decreased since 2000 (IRR of 0.97 (95% CI 0.94–1.00; *p* = 0.06)) compared to 1990–1999, the rate amongst individuals aged 18–50 has significantly increased (IRR 1.41 (95% CI 1.27–1.57; *p* < 0.001)) during the same reference time period. Although noted in both sexes, the rate of increase in incidence was significantly greater in males (11.5 to 19.7/100,000; *p* < 0.001). The overall survival from adenocarcinomas across all subsites improved in the >50-year cohort in the last decade (HR 0.89 (95% CI 0.86–0.93; *p* < 0.001)) compared to 1990–1999. In individuals aged 18–50 years, there has only been a significant improvement in survival for colorectal cancer (HR 0.82 (95% CI 0.68–0.99; *p* < 0.04)), but not the other subsites. A lower overall survival was noted for males in both age cohorts (18–50 years—HR 1.24 (95% CI 1.09–1.13; *p* < 0.01) and >50 years—HR 1.13 (95% CI 1.10–1.16; *p* < 0.001), respectively) compared to females. Conclusions: This study from South Australia demonstrates a significant increase in young-onset gastrointestinal adenocarcinomas over the last 28 years, with a greater increase in the male sex. The only significant improvement in survival in this cohort has been noted in colorectal cancer patients.

## 1. Introduction

Globally, there have been several reports of increasing incidence of early- or young-onset cancers [1]. These encompass a spectrum of solid organ cancers such as colorectal cancer (CRC) and adenocarcinomas of the pancreas [2], breast [3], ovary [4], oesophagus [5], and stomach [6]. Though CRC has demonstrated the most striking trend, similar patterns have been uncovered in other gastrointestinal adenocarcinomas. In an effort to explain this disturbing trend, clinicians and scientists have attempted to implicate early life exposures to antibiotics [7], the impact of the gut microbiome [8], and variations in mismatch repair (MMR) genes and microsatellite instability (MSI) [9]. Siegel et al. [10] linked the trend to birth cohorts and the possible influence of the obesity pandemic (drawing on the evidence relating obesity, unhealthy eating habits, and sedentary lifestyles [11]), whilst Lui et al. [12] postulated the role of lifestyle factors such as physical inactivity and alcohol consumption in the development of early onset cancers. We proposed a hypothesis, with supporting evidence [13], drawing attention to the significant contribution of perinatal events [1] drawing on the work of Barker [14], Knudson [15], and Lahouel [16]. It was postulated [1,13] that an ‘*in utero*’ insult to the foetus constitutes the ‘first hit’. The second hit would then be the result of exposures occurring in childhood and adolescence.

Koczwara et al. [17] have recently demonstrated that the coexistence of comorbidities (diabetes mellitus, chronic pulmonary disease, cardio- and cerebrovascular diseases, and peptic ulcer disease) alongside a cancer diagnosis significantly worsens survival in younger individuals. This concerning observation strengthens the relationship between the ‘developmental origins of health and disease (DOHaD)’ [14] and our hypotheses [1,13,18]. It is, thus, imperative that the cause for young-onset carcinogenesis is further investigated with the aim of detecting, and hopefully correcting, the underlying factor(s) adversely affecting survival in this cohort. In South Australia, trends in the incidence, and/or survival, of young-onset adenocarcinomas affecting abdominal viscera have not been determined. South Australia is one of the six states of Australia. It is inhabited by a population of 1.77 million that is predominantly Caucasian (>80%) and reflective of diversity seen in European countries.

The aim of this study was to determine trends in the incidence and survival of gastrointestinal adenocarcinomas (oesophagus, stomach, pancreas, and colorectal) in South Australia over a 27-year period. Being empowered with this information will offer us the opportunity to determine if data generated on young-onset cancer from global research can be extrapolated to our population. It will also enable us to join the global efforts in deciphering the causes that underpin the development of young-onset adenocarcinomas, offering South Australians and Australians, at large, the hope of improving outcomes in cancer.

## 2. Methods

We performed a cross-sectional analysis of a prospective longitudinal database to include all cases of adenocarcinoma of the oesophagus, stomach, pancreas, colon, and rectum reported to the South Australian Cancer Registry since 1 January 1990, to the latest available date at the time of our analysis, 31 December 2017. All cases of invasive cancer are notifiable under the Cancer Reporting Regulations under the *South Australian Health Care Act 2008* [19]. The South Australian Cancer Registry was established in 1977. It is managed by Wellbeing SA’s Epidemiology Branch (under the auspices of SA Health). The registry has several processes that enhance the quality of the data collection, such as electronic notification, a series of internal data checks prior to reporting the data, notification from multiple sources, and an annual internal deduplication procedure. Several features inbuilt in the Registry Plus software system also allow real time query. Statistics on quality measures can be found in the annual South Australian Cancer Registry reports [20]. The South Australian Cancer Registry has been able to completely capture all cancers across the time frame contributing to the strength of the data source.

Ethics approval for the study was obtained from the South Australian Department for Health and Wellbeing Human Research Ethics Committee (HREC) (reference number: LNR/21/SAC/51). Since only de-identified data was provided to us by the South Australian Cancer Registry, a waiver of consent was provided by the Ethics Committee.

### 2.1. Selection of Cases

#### 2.1.1. Inclusion Criteria 

All individuals who were aged 18 years and over and had a pathologically confirmed diagnosis of adenocarcinoma (ICD 10 codes: oesophagus = C15, stomach = C16, pancreas = C25, colon = C18, rectosigmoid junction = C19, rectum = C20). Histology codes for adenocarcinoma: ICD 8140/2, 8140/3, 8141/3, 8143/3, 8210/2, 8210/3, and 8230/2 and diagnosed in SA from 1 January 1990 to 31 December 2017 were included in this study. The study period was categorised into 3 eras (1990–1999, 2000–2009, 2010–2017) to reflect incidence and survivals of cancers over time. Some of the data for pancreatic adenocarcinoma has been previously published by us [21].

#### 2.1.2. Statistical Analysis

All statistical analyses were conducted using R version 4.1.0 and Stata version 16.1. Patients’ characteristics were expressed as median and interquartile range (IQR) for skewed data. The Mann–Whitney U test was used to explore the significance of differences in patients’ age between two groups of patients. Proportions were presented as percentages of the respective denominator and were compared between groups using a standard chi-square test for association with continuity correction, where appropriate.

The incidence rates were calculated by taking the total number of cases divided by the population at risk. The rates were presented per 100,000 persons over 3 time periods for age groups 18–50 years and >50 years for each sex and cancer primary sites. A Poisson regression model was applied to examine the incidence rates between the groups of the above characteristics. The estimates were calculated using the likelihood ratio method and were expressed as incidence rate ratios (IRRs) from the Poisson model. Poisson regression model was also used to calculate the average annual percentage change.

Survival was measured from the date of cancer diagnosis to the date of death, and individuals were censored at date of loss to follow-up or census date. The census date was assigned on 31 December 2017. The South Australian Cancer Registry data are linked to the births, deaths, and marriage data once a year, in general. Cox proportional hazard models were applied to examine the survival outcomes. Sex, primary sites, and cohort era were used to explore the risk of death between two cohorts (18–50 years and >50 years). The estimates were calculated using the likelihood ratio method and were expressed as hazard ratios (HRs)—the lower the HR, the longer the survival. Proportional hazard assumption was tested by the log–log plot of survival and Schoenfeld residuals. Survival curves for patient survival were evaluated by standard Kaplan–Meier survival curves and patient cohorts were compared by log-rank test. The two-sided test was performed for all analysis, 95% confidence intervals were reported, and the level of significance was set at *p* < 0.05.

## 3. Results

### 3.1. Demographic Data and Time Trend of Reported Cases

**Figure 1:**
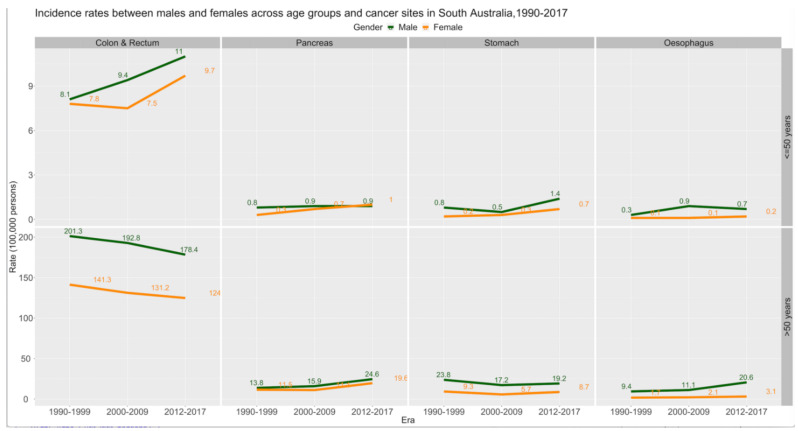
Trend in incidence rates by sex and era between two age groups across cancer sites 1990–2017 (*n* = 28,566).

**Figure 2:**
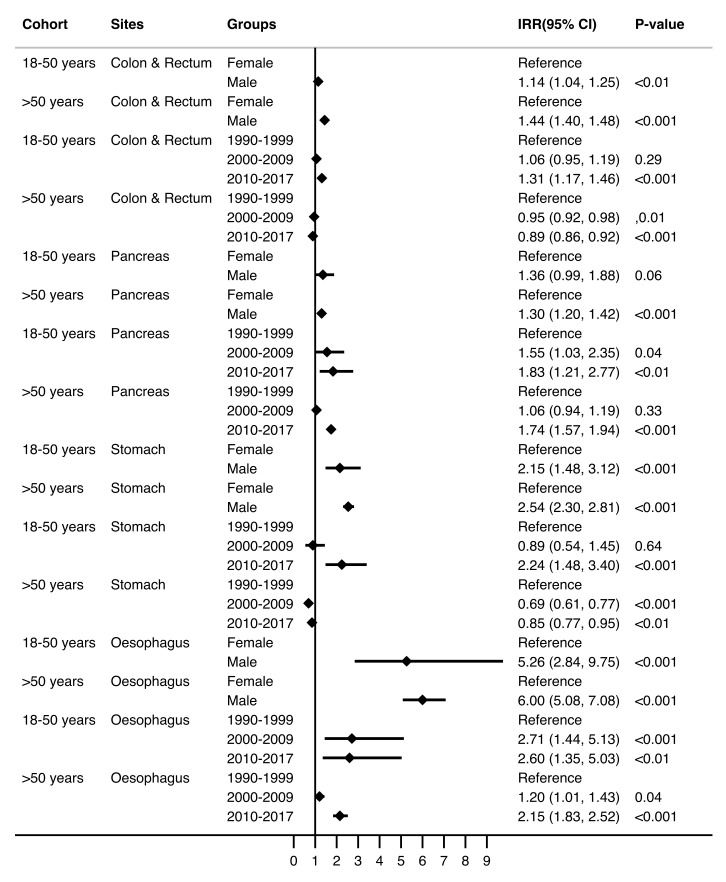
Incidence rate ratios (IRR) and 95% CI (Poisson regression model) for sex and era by primary sites between two age groups (*n* = 28,566).

A total of 28,566 patients were diagnosed with oesophageal, stomach, pancreatic, or colorectal adenocarcinoma in South Australia between 1990 and 2017 (2129, 7.5% patients aged 18–50 years and 26,437, 92.5% patients aged >50 years). The median ages for the 18–50 years and >50 years cohorts were 46 years (IQR 41–49 years) and 72 years (IQR 64–79 years), respectively (Table 1). Adenocarcinomas of the colon and rectum were the most common cancers in both age cohorts (8.85/100,000 for individuals aged 18–50 years and 159.27/100,000 for those aged >50 years). Age, in itself, was a contributing factor for incidence, with a higher increment for individuals aged 18–50 years (IRR = 1.17 (95% CI 1.16–1.18, *p* <0.001)) as compared to those >50 years (IRR 1.05 (95%CI 1.05–1.05, *p* <0.001)) (Table 2). The overall cancer incidence rates varied by sex within the two cohorts (9.42/100,000 for females and 11.78/100,000 for males aged 18–50 years and 156.19/100,000 for females and 242.33/100,000 for males >50 years). Both sexes in age cohort 18–50 years have experienced a significant increase in the incidence of adenocarcinomas (Figure 1; Supplementary Figure S1—depicting trends over 4-year intervals) over the three eras (females 8.3 to 11.9/100,000; *p* <0.001 and males 11.5 to 19.7/100,000; *p* <0.001). However, the incidence rates were significantly greater for males compared to females in both age cohorts, *viz.* 18–50 years (IRR 1.25 (95%CI 1.15–1.36; *p* <0.001)) and >50 years (IRR 1.55 (95%CI 1.51–1.59; *p* <0.001)) (Table 2). The incidence rates increased by 1% for every increment of year for males in age groups 18–50 years. However, no similar trend was noted for females in the same age group (Supplementary Table S1*)*. The sex-specific incidence rates for gastrointestinal adenocarcinomas in individuals >50 years have reduced over the three eras (Figure 1). This significant trend persisted for every cancer site across both age cohorts (Figure 2, Supplementary Table S2).

### 3.2. Trends in the Incidence of Gastrointestinal Adenocarcinomas

While the overall incidence rate for gastrointestinal adenocarcinomas in individuals >50 years in South Australia has reduced over the last three decades from 1990–1999 to 2010–2017 (203.04/100,000 to 197.16/100,000), the rates amongst individuals aged 18–50 significantly increased over the same time period from (9.13/100,000 to 12.89/100,000) (Table 2). This was confirmed by the significantly increasing trend in the IRR of 1.41 (95% CI 1.27–1.57; *p* < 0.001) in individuals aged 18–50 years, compared to a declining trend in the IRR of 0.97 (95% CI 0.94–1.00; *p* = 0.06) in those >50 years. While the IRRs for colorectal and stomach cancer significantly decreased (0.89, 95% CI 0.86–0.92; *p* < 0.001 and 0.85, 95% CI 0.77–0.95; *p* < 0.01) in the most recent decade, for the cohort aged >50 years, the IRRs for pancreatic and oesophageal cancer demonstrated a significantly increased trend (1.74, 95% CI 1.57–1.94; *p* < 0.001 and 2.15, 95% CI 1.83–1.52; *p* < 0.001, respectively). For the young-onset cohort, however, every cancer site demonstrated a significantly increased trend in the IRR (oesophagus, 2.60, 95% CI 1.35–5.03; *p* < 0.01; stomach, 2.24, 95% CI 1.48–3.40; *p* < 0.001; pancreas, 1.83, 95% CI 1.21–2.77; *p* < 0.01, and colon and rectum, 1.31, 95% CI 1.17–1.46; *p* < 0.001) (Figure 2, Supplementary Table S2).

### 3.3. Survival by Time Trends and Site 

**Figure 3:**
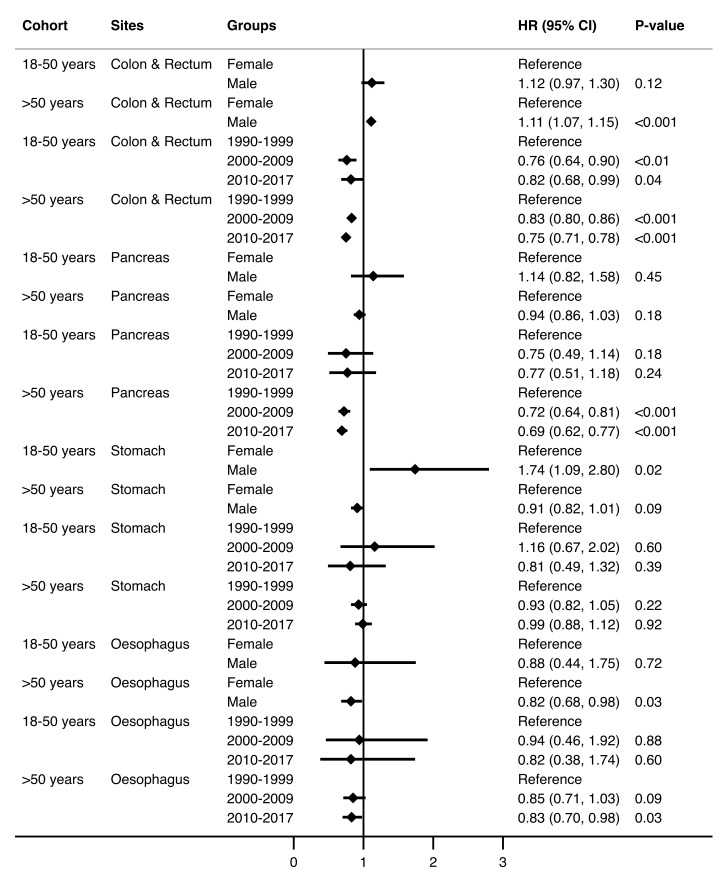
Hazard ratios (HR) and 95% CI (Cox proportional hazard model) for sex and era by primary sites between two age groups (*n* = 27,855).

The Kaplan–Meier survival estimates showed the greatest median survivals for colorectal cancer in both age cohorts (25.86 for individuals aged 18–50 years and 7.00 years for those >50 years (Table 3), and these were significantly better than the reference (oesophageal adenocarcinoma) (HR 0.28 (95% CI 0.22–0.37; *p* < 0.001) for those aged 18–50 years, and 0.34 (95% CI 0.31–0.36; *p* < 0.001) for those >50 years, respectively) (Table 4, Supplementary Figure S2). The longest median survival for colorectal cancer in the 18–50 years cohort was due to those patients diagnosed with colorectal cancer in the first half of the 1990–1999 era. The overall survival from adenocarcinomas across all subsites has improved significantly for the age cohort >50 years. However, despite demonstrating a trend in improvement, the result was not statistically significant for individuals in the 18–50 years cohort in the last decade (HR 0.92 (95% CI 0.79–1.08; *p* = 0.32)). The overall survival in males is significantly lower compared to females in both age cohorts (HR 1.24 (95% CI 1.09–1.40; *p* < 0.01) and 1.13 (95% CI 1.10–1.16; *p* < 0.001), respectively). This latter observation was largely the effect of stomach cancer (HR 1.74 (95% CI 1.09–2.80; *p* = 0.02)) in individuals aged 18–50 years and colorectal cancer (HR 1.11 (95% CI 1.07–1.15; *p* < 0.001)) in those aged >50 years (Figure 3, Supplementary Table S3). Males aged >50 years had a significantly better survival for oesophageal adenocarcinoma compared to females (HR 0.82 (95% CI 0.68–0.98; *p* = 0.03)). Reassuringly, the survival of individuals >50 years affected by most adenocarcinomas has significantly improved in the most recent decade (oesophagus, 0.83, 95% CI 0.70–0.98; *p* = 0.03; pancreas, 0.69, 95% CI 0.62–0.77; *p* < 0.001, and colon and rectum, 0.75, 95% CI 0.71–0.78; *p* < 0.001). However, in individuals aged 18–50 years, there has only been a significant improvement in survival following colorectal cancer (HR 0.82 (95% CI 0.68–0.99; *p* = 0.04)) (Figure 3, Supplementary Table S3).

## 4. Discussion

This study from South Australia demonstrates a rising incidence of young-onset (18–50 years) oesophagus, stomach, pancreas, and colorectal adenocarcinomas over a 28-year period despite a declining overall trend for individuals >50 years. In both age cohorts, the incidence rate ratio is significantly greater in males compared to females. The overall survival from adenocarcinomas across all subsites has improved significantly for the age cohort >50 years. In individuals aged 18–50 years, there has only been a significant improvement in survival for colorectal cancer, but not the other subsites.

This study presents a disturbing trend in the incidence of young-onset gastrointestinal adenocarcinomas in South Australia mirroring international data [10,12,22,23]. The change in incidence rates in younger individuals appears to be greatest for colorectal adenocarcinoma. A similar finding was previously reported by Feletto et al. [24] when studying cancers of the colon and rectum in Australia from 1982 to 2014 and by Young et al. [25] in 2015. Though the underlying causes remain to be deciphered, a similar trend in international studies has been postulated to be due to increases in early-life antibiotic use, obesity, the consumption of processed foods and alcohol [10,26,27], as well as an increase in metabolic disease (especially obesity and type 2 diabetes mellitus) seen in this similar time frame [28,29]. The relationship between the use of antibiotics and the risk of young-onset colorectal cancer has been studied by Zhang et al. [30] They found that antibiotic use was associated with a dose-dependent risk of colorectal cancer, and the location of the cancer (most commonly, proximal colon with the use of antibiotics with an anti-anaerobic activity, although an inverse relationship was noted in the rectum) altered depending on the type of antibiotic (penicillins increased the risk of colon cancer while tetracyclines were associated with a decreased risk of rectal cancer). The significant decline in colorectal adenocarcinomas in individuals >50 years in the same period of study is most likely due to early detection and prompt management of colorectal adenomas due to more effective screening of people in this age group since 2006 [24,31].

The evidence in the literature regarding the role of sex in young-onset gastrointestinal adenocarcinoma has been mixed, with some studies reporting a greater incidence rate in males for oesophageal, stomach, and colon and rectal cancers [32,33,34], while others reporting females showing greater incidence rates for colon cancer [35]. This study has demonstrated that the incidence of young-onset gastrointestinal adenocarcinomas has significantly increased in males and females between the ages of 18 and 50 years, with the increase being more pronounced in males. Putative explanations for individual adenocarcinomas include early-onset pancreatic cancer and higher rates of smoking in males [36], and early-onset stomach cancer and increased occurrence of *Helicobacter pylori* in males [33]. In the case of South Australia, given that rates for smoking have significantly reduced [37], while the prevalence of *Helicobacter pylori* infection has either remained stable or is on the decline in Australia [38,39], these are not tenable as contributory factors to the trend being witnessed. Nevertheless, although several studies report significant findings relating to sex, the reasoning for why the incidence is higher in males could possibly reflect the contribution of male central adiposity as compared to the subcutaneous adiposity that predominates in women.

While colorectal cancer had the greatest increase in incidence in the 18–50 years cohort globally, as well as in our study, the cause for this is still unclear. Family history has always been deemed a major risk factor in the development of colorectal cancer, but its role in young-onset carcinogenesis is less well understood. Although O’Connell et al. found that 22.7% of young-onset colorectal cancer had a positive family history for the disease [40], studies by Lee et al. and Dozois et al. revealed that early-onset colorectal cancer was mostly diagnosed in patients with no familial history and no genetic risk factors [41,42]. Similarly, it has been reported that the distribution of the tumour site differs significantly between those with a family history of colorectal cancer and those without, with the proximal colon being associated with patients with a positive family history, and distal colorectum for patients with no family history [43], suggesting a different carcinogenic mechanism altogether. Mirroring these reports, Bergquist et al. [44] stated that the hereditary component of stomach cancer only accounts for a minority of young-onset stomach cancer, and Piciucchi et al. and Ntala et al. reported the same with regards to young-onset pancreatic cancer [45,46]. Hypothesized mechanisms lie in genetic susceptibilities expressed in single-nucleotide instability, somatic gene mutations and epigenetic alterations, [44] as well as environmental factors such as increased sedentary living and declining dietary quality in the past three decades [27]. Oesophageal adenocarcinoma has also been on the rise since 1990, with hypotheses focusing on the role of obesity and its significant link to the pathogenesis of Barrett’s oesophagus [47], causing proinflammatory cytokines produced by visceral fat to promote carcinogenesis. Codipilly et al. [26] raised the correlation of increasing obesity in the United States within the 40–59 year old age bracket, and the increased prevalence of Barrett’s oesophagus and gastroesophageal reflux disease.

This study demonstrates a significantly improved survival for the >50 years cohort over the last three decades. This is largely due to the improved survival of colorectal cancer alone—a finding noted by Roder et al. [48]. Current oncological treatment targets the carcinogenesis of older-onset gastrointestinal adenocarcinomas, which many investigators globally suggest differ from that of young-onset cancers. Evidence of lower survival rates amongst young-onset cancers in the literature is mixed, with some studies reporting a worse prognosis and limited response to traditional therapy [44], while others are reporting better a prognosis despite more advanced disease [49], and still others indicating similar survival outcomes as compared to their older counterparts [46]. Studies reporting poorer survival rates in the younger population attribute it to more advanced stage disease at the time of diagnosis [26], though this has not been the uniform experience [50]. On the flipside, increased survival rates may be attributed to younger patients having fewer comorbidities and, hence, better responses to chemotherapy and surgery, as well as fewer postoperative complications [46]. Some believe young-onset gastrointestinal cancers to be innately more aggressive and with a differing molecular make up to their older-onset counterparts [5,26,44,50,51]. This study adds to the growing evidence [52,53] of poorer survival amongst males compared to females with young-onset cancers. Sex differences in health outcomes are increasingly being identified and studied. Sex differences in health outcomes have been shown to start in utero. A meta-analysis of RNA sequencing data in fifteen human tissues, including five brain regions, showed differential autosomal and sex chromosome gene expression between males and females in the brain, heart, kidney, colon, and thyroid, and to a lesser extent in bladder, liver, lungs, and pancreas [54]. These may underpin the sexually dimorphic incidence and survival for various cancers.

The rise in incidence of young-onset gastrointestinal adenocarcinomas in South Australia raises more questions and highlights major gaps in our knowledge and understanding of causal mechanisms. It emphasizes the need to explore the environmental and behavioural factors during early life. Consideration of the contribution of perinatal and early-life events in the development of young-onset carcinogenesis is of particular interest [1,13]. While lowering the age for screening programs could result in earlier detection, currently screening in Australia only exists for colorectal cancer for individuals over 50 years [31]. In the case of Barrett’s oesophagus, South Australia has a well-established surveillance programme [55,56]. Increased screening carries with it a new set of challenges. The American College of Gastroenterology Guidelines for Colorectal Cancer Screening 2021 [57] recommends the initiation of screening at 45 years instead of 50 years for average-risk individuals, reducing colorectal cancer risk due to earlier detection, and the removal of polyps. It was believed this would reduce the incidence of colorectal cancer in those >50. Yet, a calculated additional 21 million individuals aged 45–49 would need to be screened yearly, creating a significant burden on an already overloaded healthcare system [57]. In South Australia, screening begins at 50 years of age, and patients with a positive faecal immunochemical test (FIT) must undergo colonoscopy within 3 months. Were the screening to drop to 45 years of age, an already strained healthcare system would have to deal with a surge in the requests for colonoscopy with a lack of a clear benefit and would thus present a challenge. As it is unlikely to be a viable or long-term solution, it is imperative we address the factors underlying the rising incidence of young-onset cancers.

One of the major strengths of this study has been the strict inclusion criteria that enabled us to select only oesophageal, stomach, pancreatic, and colorectal adenocarcinoma patients in the past three decades. This may explain the very clear trajectory noted in the studied cancers compared to previous papers from Australia that included all types of cancers affecting a subsite [58,59]. Another strength of this study lies in the appreciation that analysing annual or even four-yearly (Supplementary Figure S1 and Supplementary Table S1) trends in the incidence and survival of diseases, such as cancer, that occur at a lower incidence in smaller populations is fraught with the risk of overlooking important variations over time. The decision to analyse the data in longer time cohorts enabled us to demonstrate the true magnitude of the problem in our region. The data are derived from the South Australian Cancer Registry, a long-standing cancer registry where pathology, death, and clinical reporting is mandatory by law. The advantage of this is rigorous processes of collection of high-quality data. However, it lacks the details of treatment and the stage of the disease at diagnosis. Moreover, the Registry does not contain detailed socio-economic data. These data (on disease stage and sociodemographic variables of race, education, income, etc.) are invaluable to determine the factors involved in the causation of this emerging problem of the rising incidence of young-onset cancers. Being able to decipher the underlying factors will not only reveal any disparities in trends based on demographics, but it will also inform us of the strategies to be employed to prevent the development of these cancers. Nonetheless, this data is invaluable in providing a real-world analysis of young-onset gastrointestinal adenocarcinomas in South Australia.

## 5. Conclusions

This study from South Australia demonstrates a significant increase in young-onset gastrointestinal adenocarcinomas over the last 28 years, with a greater increase in the male sex. The only significant improvement in survival in this cohort has been noted in colorectal cancer patients. These results signal the need for a concerted global effort in deciphering the causes that underpin the development of young-onset adenocarcinomas, offering the hope of improving outcomes in young cancer patients.

## Figures and Tables

**Table 1:** Patient’s characteristics, era, and primary sites of cancer between two age groups (N = 28,566).

Patient Characteristics	(18–50 Years)		(>50 Years)		
	*n* = 2129 (7.5%)		*n* = 26,437 (92.5%)		
	*n*	%	*n*	%	*p*-Value
Age (years): median ± IQR	46	(41–49)	72	(64–79)	<0.001
Sex					0.09
Female	1190	55.9	15275	57.8	
Male	939	44.1	11162	42.2	
Era					0.49
1990–1999	650	30.5	7994	30.2	
2000–2009	720	33.8	9270	35.1	
2010–2017	759	35.7	9173	34.7	
Primary site					0.058
Colon and Rectum	1776	83.4	21422	81.0	
Pancreas	150	7.0	2163	8.2	
Stomach	127	6.0	1808	6.8	
Oesophagus	76	3.6	1044	3.9	

Note. Number and percentages are reported unless stated otherwise; IQR—interquartile range. The *p* values are based on Mann–Whitney U test for medians and chi-square test for proportions.

**Table 2:** Incidence rates (IR) and incidence rate ratios (IRR) for age, sex, era, and cancer sites between two age groups (*n* = 28,566).

Patient Characteristics	(18–50 Years)			(>50 Years)		
	*n* = 2129			*n* = 26,437		
	* IR (95% CI)	IRR (95% CI)	*p*-Value	* IR (95% CI)	IRR (95% CI)	*p*-Value
Overall	10.60 (10.16–11.06)			196.56 (194.20–198.94)		
Age (years)	-	1.17 (1.16–1.18)	<0.001	-	1.05 (1.05–1.05)	<0.001
Sex						
Female	9.42 (8.82–10.04)	Reference	-	156.19 (153.30–159.11)	Reference	-
Male	11.78 (11.12–12.46)	1.25 (1.15–1.36)	<0.001	242.33 (238.51–246.21)	1.55 (1.51–1.59)	<0.001
Era						
1990–1999	9.13 (8.44–9.86)	Reference	-	203.04 (198.61–207.53)	Reference	-
2000–2009	10.19 (9.46–10.96)	1.12 (1.00–1.24)	0.04	190.74 (186.87–194.66)	0.94 (0.91–0.97)	<0.001
2010–2017	12.89 (11.98–13.83)	1.41 (1.27–1.57)	<0.001	197.16 (193.15–201.24)	0.97 (0.94–1.00)	0.06
Cancer site						
Colon & Rectum	8.85 (8.44–9.27)	-	-	159.27 (157.15–161.42)	-	-
Pancreas	0.75 (0.63–0.88)	-	-	16.08 (15.41–16.77)	-	-
Stomach	0.63 (0.53–0.75)	-	-	13.44 (12.83–14.08)	-	-
Oesophagus	0.38 (0.30–0.47)	-	-	7.76 (7.30–8.25)	-	-

* IR is incidence per 100,000 South Australian residents. IRs were not reported for age and IRRs were not reported for cancer sites.

**Table 3:** Median survival times for primary sites of cancer between two age groups (*n* = 27,855).

Cancer Sites	(18–50 Years)	(>50 Years)
	*n* = 2107 (7.6%)	*n* = 25,748 (92.4%)
	Median (95% CI)	Median (95% CI)
Overall	12.67 (9.19–17.07)	4.60 (4.44–4.77)
Colon and Rectum	25.86 (19.90-NA)	7.00 (6.81–7.24)
Pancreas	0.70 (0.56–0.86)	0.48 (0.44–0.52)
Stomach	1.32 (1.02–1.91)	0.94 (0.85–1.02)
Oesophagus	1.36 (0.87–2.54)	0.99 (0.88–1.05)

Note: Upper confidence level of survival time for colon and rectum cancer exceeded the follow-up time. NA = not available

**Table 4:** Hazard ratios (HR) and 95% CI (Cox proportional hazard model) for age, sex, era, and cancer sites between two age groups (*n* = 27,855).

Variables	(18–50 Years)		(>50 Years)	
	*n* = 2107		*n* = 25,748	
	HR (95% CI)	*p* Value	HR (95% CI)	*p* Value
Age (years)	1.01 (1.00–1.02)	0.03	1.04 (1.04–1.04)	<0.001
Sex				
Female	Reference	-	Reference	-
Male	1.24 (1.09–1.40)	<0.01	1.13 (1.10–1.16)	<0.001
Era				
1990–1999	Reference	-	Reference	-
2000–2009	0.86 (0.74–1.00)	0.046	0.85 (0.82–0.88)	<0.001
2010–2017	0.92 (0.79–1.08)	0.32	0.89 (0.86–0.93)	<0.001
Cancer site				
Colon and Rectum	0.28 (0.22–0.37)	<0.001	0.34 (0.31–0.36)	<0.001
Pancreas	2.48 (1.83–3.36)	<0.001	2.13 (1.97–2.31)	<0.001
Stomach	0.95 (0.69–1.32)	0.77	0.94 (0.86–0.36)	0.12
Oesophagus	Reference	-	Reference	-

## Data Availability

Authors are unable to provide this data owing to the Ethics approval being granted on the premise that the (South Australian Cancer Registry) data will not be released to a third party.

## Supplementary Materials

The following supporting information can be downloaded at: https://www.mdpi.com/article/10.3390/cancers14020275/s1. Table S1: Average annual percentage change, AAPC, (Poisson Regression Model) for gender and primary sites of cancer between two age groups (N = 28,566); Table S2: Incidence rate ratios (IRR) and 95% CI (Poisson regression model) for sex and era by primary sites between two age groups (n = 27,855); Table S3: Hazard ratios (HR) and 95% CI (Cox Proportional hazard model) for sex and era by primary sites between two age groups (n = 27,855). Supplementary Figure S1: Trend in incidence rates by sex and era between two age groups across cancer sites 1990–2017 (n = 28,566); Supplementary Figure S2: Kaplan-Meier survival curves for sex, era and primary sites between age groups.
