# Supplementary material for: Gastrointestinal Adenocarcinoma Incidence and Survival Trends in South Australia, 1990–2017
Source: Cancers (Basel). 2022 Jan 6;14(2):275. doi: 10.3390/cancers14020275 (PMC8773524; doi:10.3390/cancers14020275)
Supplement: Supplementary file 1 [file cancers-14-00275-s001.zip › cancers-1514322-supplementary.pdf]

Supplementary data

Table S1. Average annual percentage change, AAPC, (Poisson Regression Model) for gender and primary sites of cancer between two age groups (N=28,566).

	(18-50 years) N=2,129		(>50 years) N=26,437	
	**AAPC (95% CI)**	**P Value**	**AAPC (95% CI)**	**P Value**
**Sex**				
Female	1.00 (0.99-1.01)	0.62	1.00 (1.00-1.00)	0.10
Male	1.01 (1.00-1.02)	<0.01	1.00 (1.00-1.00)	0.58
**Cancer site**				
Colon & Rectum	1.01 (1.01-1.02)	<0.001	0.99 (0.99-1.00)	<0.001
Pancreas	1.02 (1.00-1.04)	0.07	1.03 (1.02-1.04)	<0.001
Stomach	1.04 (1.02-1.06)	<0.001	0.99 (0.99-1.00)	0.02
Oesophagus	1.01 (0.98-1.04)	0.68	1.05 (1.04-1.05)	<0.001

Table S2. Incidence rate ratios (IRR) and 95% CI (Poisson regression model) for sex and era by primary sites between two age groups (n=27,855).

	(18-50 years) N=2,107		(>50 years) N=25,748	
	**IRR (95% CI)**	**P-value**	**IRR (95% CI)**	**P-value**
Colon & Rectum				
**Sex**				
Female	Reference	-	Reference	-
Male	1.14 (1.04-1.25)	<0.01	1.44 (1.40-1.48	<0.001
**Era**				
1990-1999	Reference	-	Reference	-
2000-2009	1.06 (0.95-1.19)	0.29	0.95 (0.92-0.98)	<0.01
2010-2017	1.31 (1.17-1.46)	<0.001	0.89 (0.86-0.92)	<0.001
Pancreas				
**Sex**				
Female	Reference	-	Reference	-
Male	1.36 (0.99-1.88)	0.06	1.30 (1.20-1.42)	<0.001
**Era**				
1990-1999	Reference		Reference	
2000-2009	1.55 (1.03 – 2.35)	0.04	1.06 (0.94-1.19)	0.33
2010-2017	1.83 (1.21-2.77)	<0.01	1.74 (1.57-1.94)	<0.001
Stomach/Intestine				
**Sex**				
Female	Reference	-	Reference	-
Male	2.15 (1.48-3.12)	<0.001	2.54 (2.30-2.81)	<0.001
**Era**				
1990-1999	Reference	-	Reference	-
2000-2009	0.89 (0.54-1.45)	0.64	0.69 (0.61-0.77)	<0.001
2010-2017	2.24 (1.48-3.40)	<0.001	0.85 (0.77-0.95)	<0.01
Oesophagus				
**Sex**				
Female	Reference	-	Reference	-
Male	5.26 (2.84-9.75)	<0.001	6.00 (5.08-7.08)	<0.001
**Era**				
1990-1999	Reference	-	Reference	-
2000-2009	2.71 (1.44-5.13)	<0.01	1.20 (1.01-1.43)	0.04
2010-2017	2.60 (1.35-5.03)	<0.01	2.15 (1.83-2.52)	<0.001

Table S3. Hazard ratios (HR) and 95% CI (Cox Proportional hazard model) for sex and era by primary sites between two age groups (n=27,855).

	(18-50 years)		(>50 years)	
	N=2,107		N=25,748	
	**HR (95% CI)**	**P-value**	**HR (95% CI)**	**P-value**
Colon & Rectum				
**Sex**				
Female	Reference	-	Reference	-
Male	1.12 (0.97-1.30)	0.12	1.11 (1.07-1.15)	<0.001
**Era**				
1990-1999	Reference	-	Reference	-
2000-2009	0.76 (0.64-0.90)	<0.01	0.83 (0.80-0.86)	<0.001
2010-2017	0.82 (0.68-0.99)	0.04	0.75 (0.71-0.78)	<0.001
Pancreas				
**Sex**				
Female	Reference	-	Reference	-
Male	1.14 (0.82-1.58)	0.45	0.94 (0.86-1.03)	0.18
**Era**				
1990-1999	Reference	-	Reference	-
2000-2009	0.75 (0.49-1.14)	0.18	0.72 (0.64-0.81)	<0.001
2010-2017	0.77 (0.51-1.18)	0.24	0.69 (0.62-0.77)	<0.001
Stomach/Intestine				
**Sex**				
Female	Reference	-	Reference	-
Male	1.74 (1.09-2.80)	0.02	0.91 (0.82-1.01)	0.09
**Era**				
1990-1999	Reference	-	Reference	-
2000-2009	1.16 (0.67-2.02)	0.60	0.93 (0.82-1.05)	0.22
2010-2017	0.81 (0.49-1.32)	0.39	0.99 (0.88-1.12)	0.92
Oesophagus				
**Sex**				
Female	Reference	-	Reference	-
Male	0.88 (0.44-1.75)	0.72	0.82 (0.68-0.98)	0.03
**Era**				
1990-1999	Reference	-	Reference	-
2000-2009	0.94 (0.46-1.92)	0.88	0.85 (0.71-1.03)	0.09
2010-2017	0.82 (0.38-1.74)	0.60	0.83 (0.70-0.98)	0.03

Figure S1. Trend in incidence rates by sex and era between two age groups across cancer sites 1990-2017 (n=28,566).

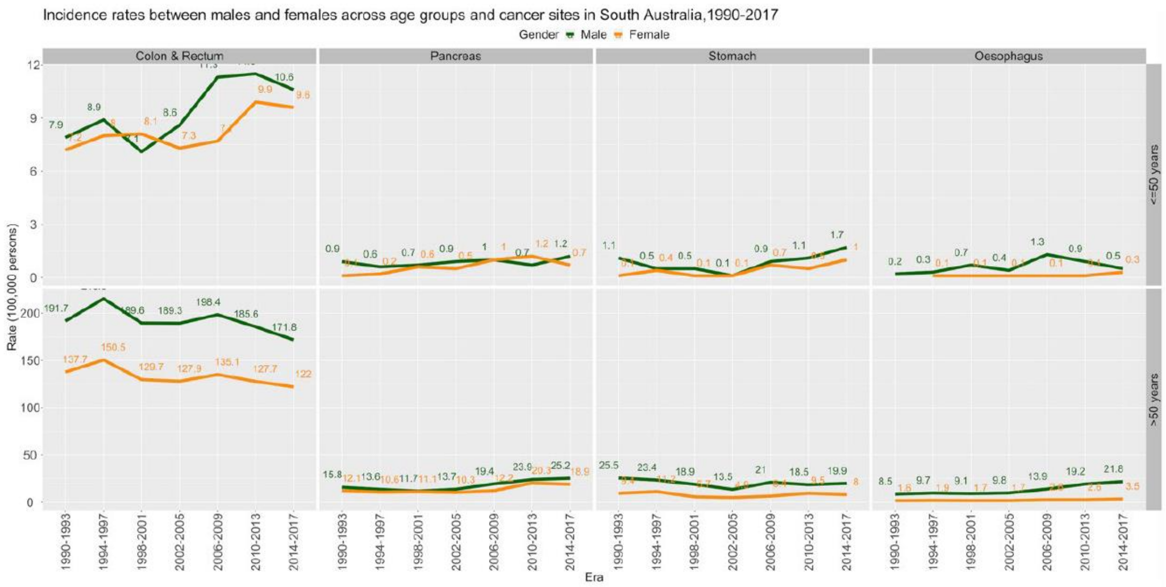

Figure S2. Kaplan-Meier survival curves for sex, era and primary sites between age groups

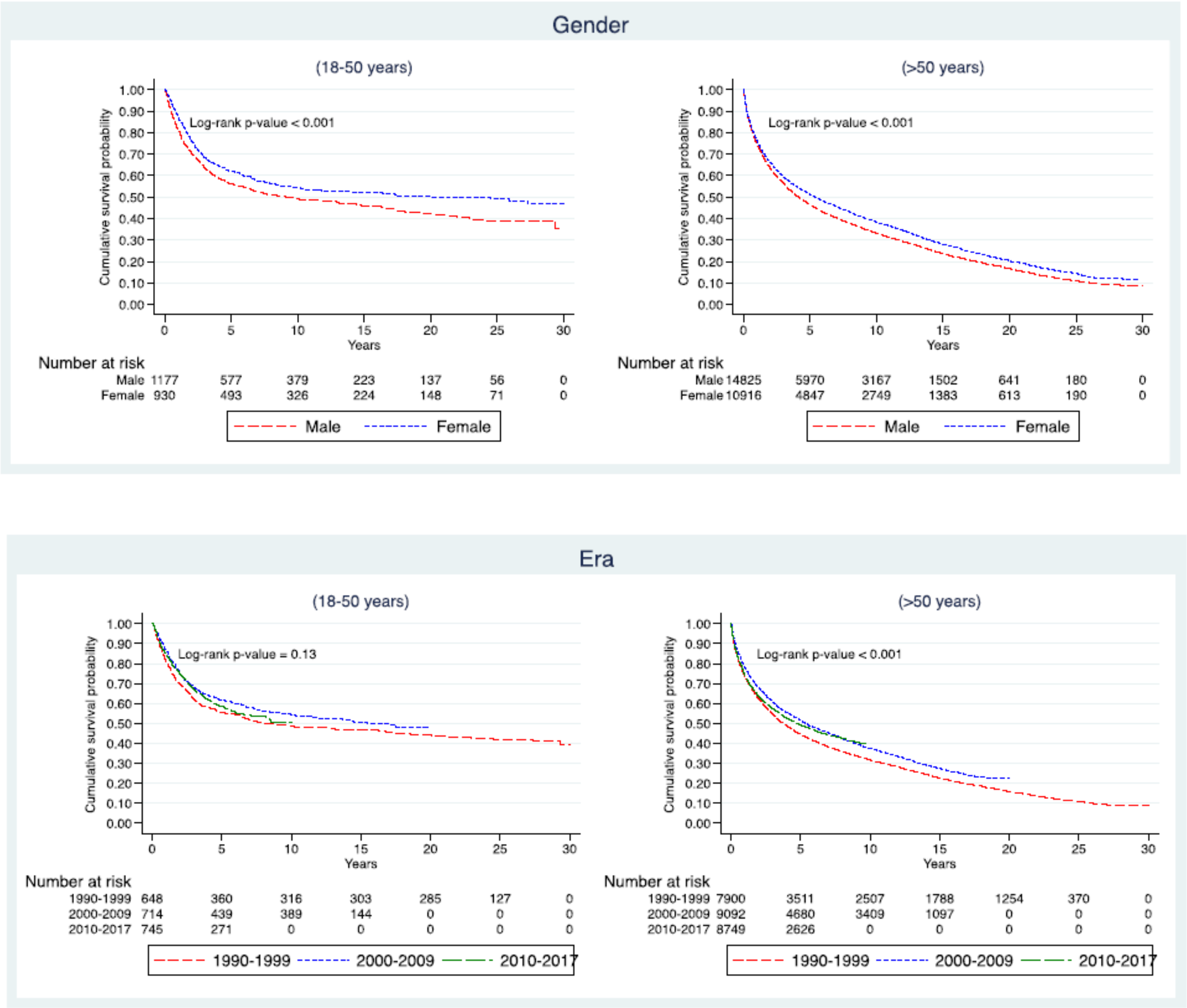

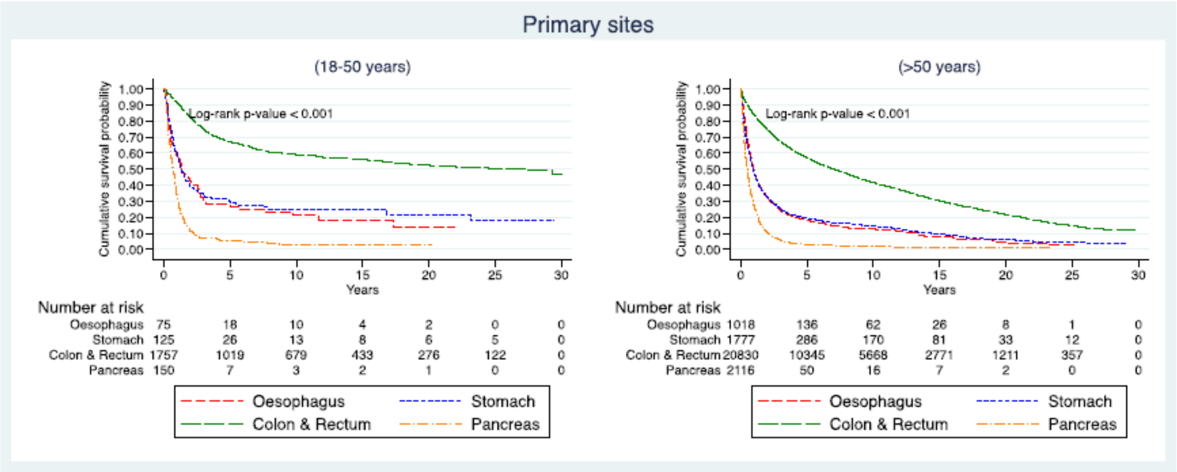
Primary sites
(18-50 years)
Log-rank p-value < 0.001
Cumulative survival probability
1.00
0.90
0.80
0.70
0.60
0.50
0.40
0.30
0.20
0.10
0.00
0
5
10
15
20
25
30
Years
Number at risk
Oesophagus 75 18 10 4 2 0 0
Stomach 125 26 13 8 6 5 0
Colon & Rectum 1757 1019 679 433 276 122 0
Pancreas 150 7 3 2 1 0 0
Oesophagus
Stomach
Colon & Rectum
Pancreas
(>50 years)
Log-rank p-value < 0.001
Cumulative survival probability
Years
Number at risk
Oesophagus 1018 136 62 26 8 1 0
Stomach 1777 286 170 81 33 12 0
Colon & Rectum 20830 10345 5668 2771 1211 357 0
Pancreas 2116 50 16 7 2 0 0
Oesophagus
Stomach
Colon & Rectum
Pancreas
